# Barriers to the management of sexual dysfunction among people with psychosis: analysis of qualitative data from the REMEDY trial

**DOI:** 10.1186/s12888-022-04193-7

**Published:** 2022-08-12

**Authors:** Lavanya J. Thana, Lesley O’Connell, Alexandra Carne-Watson, Abhishek Shastri, Arunan Saravanamuthu, Natasha Budhwani, Sandra Jayacodi, Verity C. Leeson, Jasna Munjiza, Sofia Pappa, Elizabeth Hughes, Joe Reilly, Mike J. Crawford

**Affiliations:** 1 Division of Psychiatry, Commonwealth Building, Imperial College London, Du Cane Road, London, W12 0NN UK; 2 Tees, Esk and Wear Valleys NHS Foundation Trust, Darlington, UK; 3 Central and North West London NHS Foundation Trust, London, UK; 4 West London NHS Health Trust, London, UK; 5 School of Health and Social Care, Edinburgh Napier University, Edinburgh, UK

**Keywords:** Psychotic disorders, Antipsychotic medication, Sexual dysfunction

## Abstract

**Background:**

More than half of people who use antipsychotic medication for psychosis report having sexual dysfunction. The REMEDY trial aimed to find out if switching antipsychotic medication provides an effective way to reduce sexual dysfunction among people with psychosis. We set out to recruit 216 participants over a two-year period, but recruitment was stopped after an extended 12-month pilot phase, during which we recruited only 10 participants. As part of a nested process evaluation, we conducted qualitative interviews with front-line clinicians to examine barriers to recruitment to the trial.

**Methods:**

We developed a semi-structured interview schedule to explore staff views on factors that influenced whether they referred potential participants to the study. We interviewed a purposive sample of 51 staff from four National Health Service (NHS) Trusts in England, ensuring a range of different backgrounds, seniority, and levels of involvement in the trial. Audio recordings of interviews were transcribed for verbatim, and data were analysed using an inductive approach to thematic analysis.

**Results:**

Nine interconnected themes were generated. Six themes concerned barriers to recruitment; including; prioritising patients’ mental stability, mutual discomfort and embarrassment about discussing a “taboo” subject, and concerns about unintended consequences of asking people with psychosis about their sexual functioning. Three themes, including the quality of treatment relationships and strategies for opening dialogue suggested ways to improve recognition of these “hidden” side effects.

**Conclusion:**

The identification and management of sexual dysfunction among people with psychosis are not priorities for mental health services in England at this time. Many staff working in front-line services feel unprepared and uncomfortable asking people with psychosis about these problems. While greater use of screening tools may improve the identification of sexual dysfunction among people with psychosis, the evaluation and implementation of interventions to manage them will continue to be challenging unless NHS leaders and senior clinicians demonstrate greater commitment to changing current clinical practice.

**Trial registration:**

Current Controlled Trials ISRCTN12307891.

## Background

Antipsychotic medication can improve the mental health of most people with psychosis, but it can also cause side effects that negatively impact on people’s health and quality of life. At least half of people who take antipsychotic medication report sexual dysfunction [1–3]. Most antipsychotic medications block dopamine receptors which can lead to increased serum prolactin levels and reduced sexual arousal (erectile dysfunction in men and reduced vaginal lubrication in women) and libido [4]. Antipsychotic drugs can also impair sexual functioning through sedation and by direct effects on blood flow to reproductive organs [5, 6].

Sexual expression is an important aspect of human experience and impaired sexual functioning can therefore have a major impact on a person’s quality of life [7]. People with psychosis who experience sexual dysfunction also report lower quality of life and are less likely to have an intimate relationship [8, 9]. People with psychosis who do have an intimate relationship, rate the quality of the quality of their relationship lower if they are experiencing sexual dysfunction [10]. People with psychosis who have sexual dysfunction, often attribute this to the medication that they take [11]. Sexual dysfunction can lead to poor adherence with antipsychotic medication. Surveys of people with psychosis indicate that sexual dysfunction is a common reason for stopping antipsychotic medication, resulting in an increased likelihood of relapse and readmission to hospital [11–13]. Given that sexual dysfunction associated with use of antipsychotic medication can have a major impact on quality of life, risk of relapse and cost of care, it is vital that it is assessed and effectively managed to minimise or prevent these negative outcomes.

Considerable efforts have gone into developing and evaluating interventions for managing common side effects of antipsychotic medications such as weight gain and movement disorders. In contrast, research into the management of sexual dysfunction associated with these drugs is limited [14]. Research aimed at developing better management of sexual dysfunction associated with antipsychotic drugs has been identified as a national research priority by people who use and provide services for people with psychosis [15].

One treatment option available to clinicians for trying to manage these side effects is to consider switching antipsychotic medication to one that is less likely to impact on sexual functioning [16]. However, a change in medication may pose risks to mental stability as well as result in other unwanted side effects [17, 18]. Only limited small-scale studies have attempted to assess the effect of switching antipsychotic medication for the purpose of reducing sexual dysfunction associated with use of these medications [19, 20] and these are insufficient to inform clinical practice [14, 21].

To improve the evidence base on the management of sexual dysfunction associated with the use of antipsychotic medication, the National Institute for Health Research (NIHR) in England, commissioned the REMEDY trial (Randomised Evaluation of Management of sExual DYsfunction) [22]. The main aim of the trial was to try to find out if switching to an alternative antipsychotic with a lower potential of impacting on sexual functioning, provides a safe and effective strategy for improving sexual functioning among people with psychosis. We set out to recruit 216 participants over a 21-month period from four large Mental Health Trusts in England. However, after an extended 12-month pilot phase of the trial, only 10 participants had been recruited. A decision was therefore made to stop the study prematurely. The REMEDY trial included plans for a nested process evaluation designed to examine the implementation of the trial and the impact of context on study outcomes [23]. However, as the study progressed, we realised how challenging recruitment was and changed the focus of the qualitative component of the study instead to examine barriers to recruitment to the study [22].

## Methods

A detailed description of the trial’s design, methods, and results have been reported elsewhere [22]. A summary of the methods are outlined below followed by a description of the qualitative methods and approach to analysing these data. An independent Trial Steering Committee and Data Monitoring and Ethics Committee oversaw the trial.

### Trial design

The REMEDY trial was a two-arm, parallel-group, researcher-blind, randomised trial with an internal pilot. To take part in the study, potential participants needed to be aged 18 years or over, be in contact with secondary mental health services, have a clinical diagnosis of schizophrenia and related psychoses, and report sexual dysfunction associated with the use of antipsychotic medication. Potential participants also had to meet the criteria for significant sexual dysfunction on the Arizona Sexual Experience scale [24, 25]. People were only recruited to the study if a reduction in the dose of their current antipsychotic medication was deemed ineffective or clinically inappropriate.

Participants were randomised to either a switch in medication plus enhanced standard care or enhanced standard care alone. For those in the active arm of the trial, a switch to aripiprazole, quetiapine or olanzapine was agreed based on the participants’ previous response to antipsychotic medication, the side effect profile of these drugs, and patient preference. All study participants received enhanced treatment as usual. This comprised of usual care plus up to two sessions of brief psychoeducation and support to discuss sexual health and functioning.

Mental health staff working across inpatient and community-based services were asked to identify potential participants either during clinical consultations, or by reviewing existing clinical records.

During the pilot phase of the trial, we aimed to recruit 36 participants across three NHS Trusts in England over a six-month period. Having recruited only six participants, an agreement was reached with the funder to extend the pilot phase of the trial by a further six months. A further four participants were recruited during this period and the trial was halted.

### Collection of qualitative data

Staff working in the four recruitment sites and who had been involved in REMEDY recruitment were invited to participate in a semi-structured interview with a study researcher during the recruitment phase of the trial or in the six months following the end of recruitment. We selected a purposive sample of NHS staff to ensure maximum variation in the perspectives and experiences of people from diverse backgrounds and seniority. In consultation with an ‘Expert Advisory Panel’ of people with lived experience of poor mental health, a semi-structured interview schedule was developed to explore staff views on factors that promoted and hindered referrals to the study. Face-to-face interviews were audio-recorded and lasted for up to one hour. Recordings were professionally transcribed and then quality checked by the research team before coding and data analysis.

### Data analysis

Interview data were analysed using an interpretative approach to Thematic Analysis [26]. The researcher (LT) familiarised herself with the data and inductively generated codes and themes concerning contextual factors, mechanisms affecting recruitment failure and associations that were overtly stated as well as those that may be inherent in the interviewee’s descriptions, but not consciously put forward by participants. LT was an independent and experienced qualitative research associate, working on other mental health-related projects at the time. She was aware that the research team encountered difficulty in recruiting participants but little else due to her external capacity. MC reviewed the developing analysis at various interim stages. He was the chief investigator of the trial with expertise in mixed methods research.

Interim findings from analysis of the first 11 interviews were presented to members of the Trial Management Committee. Following this, minor changes were made to the content of the interview guides and questions added to take account of feedback from the group as well as to explore developing themes in greater depth. The remaining interviews were conducted using the amended guides. Although the notion of data saturation is contested by various non-positivist qualitative scholars [26] and was not an analytical aim of this work, the large sample enabled elements of higher-order themes as well as relationships between codes and themes to be ‘sufficiently’ and confidently developed [27, 28].

The generated themes were scrutinised, refined, and restructured as necessary, and presented for review by the Expert Advisory Panel for ‘member-checking’ before finalising. These finalised themes are presented below.

## Results

Fifty-one members of staff took part in the qualitative component of the study. The demographic and professional backgrounds of these staff are presented in Table 1. Staff represented a broad range of professions, experience, and demographics. Just over half of respondents were psychiatrists (*n* = 27, 52.9%). Experience ranged from junior trainees to consultant grade psychiatrists. Sixteen (31.4%) participants were mental health nurses, three were peer support workers and the remaining two were social workers. Staff highlighted the complexity, and thus difficulty, of recruiting patients to the trial. Many reported that they had not referred patients to the trial despite being aware of ongoing recruitment efforts in their respective sites. The following themes elucidate the challenges they experienced.

**Table 1:** Demographic and professional details of staff participants

Identification number	Sex	Ethnicity	Professional background	Job title	Time spent working in mental health
1ST001	Female	White—other	Doctor	Consultant Psychiatrist	10–15 years
1ST002	-	-	Doctor	Psychiatrist	10–15 years
1ST003	-	-	Doctor	Psychiatrist	> 15 years
1ST004	Male	-	Doctor	Psychiatrist	10–15 years
1ST005	Male	-	Doctor	Psychiatrist	5–10 years
1ST006	-	-	Doctor	Psychiatrist	10–15 years
1ST007	Female	-	Mental Health Nurse	Mental Health Nurse	< 5 years
1ST008	Female	-	Mental Health Nurse	Community Psychiatric Nurse	10–15 years
1ST009	Female	-	Mental Health Nurse	Mental Health Nurse	< 5 years
1ST010	Female	British Asian	Mental Health Nurse	Mental Health Nurse	< 5 years
1ST011	Male	White—other	Doctor	Consultant Psychiatrist	< 5 years
1ST012	Male	Asian—other	Mental Health Nurse	Mental Health Nurse	> 15 years
1ST013	Male	Other—Chinese	Doctor	Specialist Registrar	< 5 years
1ST014	Female	White—other	Doctor	Psychiatrist	> 15 years
1ST015	Male	White—other	Doctor	Consultant Psychiatry	> 15 years
1ST016	Male	White British	Social worker	Care co-ordinator/social worker	> 15 years
1ST017	Male	Black—African	Mental Health Nurse	Mental Health Nurse	> 15 years
1ST018	Female	Mixed	Mental Health Nurse	Mental Health Nurse	> 15 years
1ST019	Female	White—other	Doctor	Psychiatrist	> 15 years
1ST020	Male	Asian—other	Doctor	Consultant Psychiatrist	> 15 years
1ST021	Male	British Asian	Mental Health Nurse	Mental Health Nurse	> 15 years
1ST022	Female	White British	Mental Health Nurse	Mental Health Nurse	< 5 years
1ST023	Male	Black African	Mental Health Nurse	Nurse Manager	> 15 years
1ST024	Female	White—other	Social Worker	Social Worker	> 15 years
1ST025	Male	White British	Mental Health Nurse	Staff Nurse	5–10 years
1ST026	Female	White British	Pharmacist	Pharmacist	> 15 years
1ST027	Female	Black British	Mental Health Nurse	Senior Staff Nurse	10–15 years
1ST028	Female	White—other	Doctor	Consultant Psychiatrist	> 15 years
1ST029	Female	British Asian	Pharmacy Technician	Pharmacy Technician	10–15 years
1ST030	Female	White—other	Doctor	Psychiatrist	< 5 years
1ST031	Female	White British	Peer Support Worker	Peer Support co-ordinator	< 5 years
1ST032	Female	Black African	Pharmacist	Pharmacist	10–15 years
1ST033	Female	White British	Peer Support Worker	Peer Worker	5–10 years
1ST034	Female	White British	Peer Support Worker	Peer Support Worker	< 5 years
2ST001	Female	British White	Doctor	Specialty Registrar	< 5 years
2ST002	Female	White—other	Doctor	Consultant Psychiatrist	> 15 years
2ST003	Male	-	Doctor	Specialty Doctor	< 5 years
2ST004	Male	British Asian	Doctor	Consultant Psychiatrist	> 15 years
2ST005	Male	-	Nurse	Senior Nurse Practitioner	-
2ST006	Male	-	Nurse	Community Psychiatric Nurse	-
2ST007	Female	Black African	Doctor	Specialty Doctor	-
3ST001	Male	White British	Doctor	Consultant Psychiatrist	> 15 years
3ST002	Female	Black African	Doctor	Consultant Psychiatrist	10–15 years
3ST003	Female	White British	Doctor	Consultant Psychiatrist	> 15 years
3ST004	Female	African	Doctor	Consultant Psychiatrist	> 15 years
3ST005	Male	White, non-British	Doctor	Consultant Psychiatrist	10–15 years
3ST006	Male	British Asian	Doctor	Psychiatry	5–10 years
3ST007	Male	White British	Nurse	Senior Care Co-ordinator	10–15 years
3ST008	Male	Indian	Doctor	Consultant Psychiatrist	> 15 years
3ST009	Female	White British	Doctor	Consultant Psychiatrist	> 15 years
3ST010	Female	White British	Mental Health Nurse	Team Manager	> 15 years

(-) = missing data when participant declined to provide this information

**Fig. 1:**
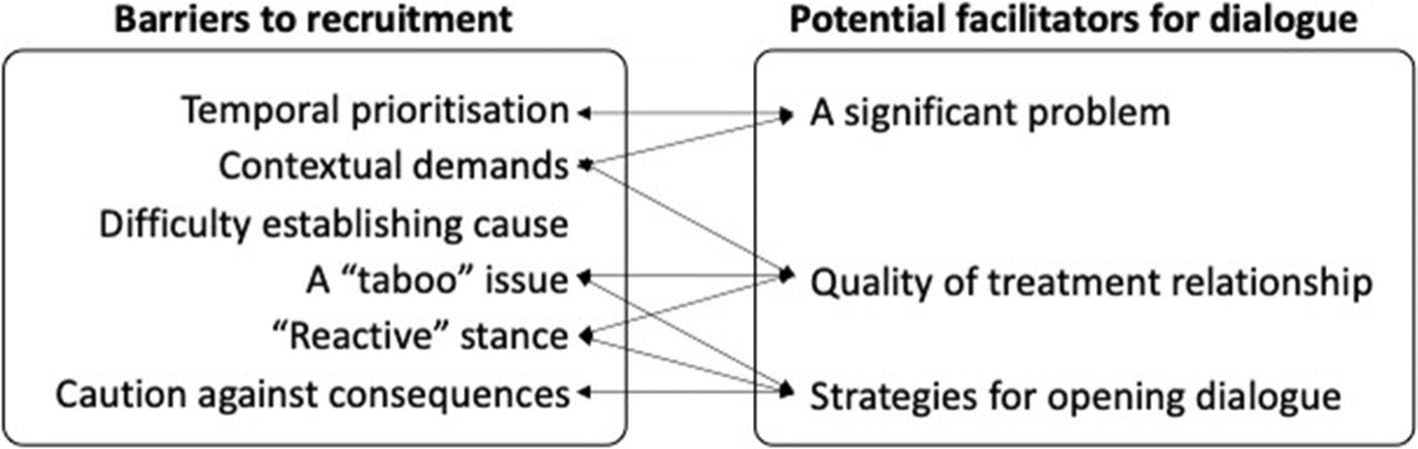
Classification of themes into barriers and facilitators to discussion of sexual dysfunction. Arrows indicate the direction of the relationships between items. Bi-directional relationships are indicated by double headed arrows

Nine themes were generated from the data. Six themes were grouped into barriers to recruitment and three into facilitating factors. Barriers to recruitment were: (1) staff feeling that the time was not right for talking to people about sexual dysfunction and the REMEDY trial, (2) staff feeling that they did not have enough time to raise this subject because of competing demands, (3) staff beliefs that patients would not want to be asked about this ‘taboo’ subject, (4) staff preference for patients to raise the issue of sexual dysfunction rather than asking them directly about it, (5) staff doubts about whether it would be possible to establish whether, if patients had sexual dysfunction, this was the result of side effects of the medication they were taking, and (6) staff concerns about unintended and potentially harmful consequences of talking to people with psychosis about sexual functioning with patients. Factors that appeared to facilitate dialogue about sexual dysfunction and the REMEDY trial were: the recognition that sexual dysfunction was associated with use of antipsychotic medication was a significant problem, (2) the need for a conducive setting and time to enable staff to build trust and rapport with patients, and (3) using strategies to approach this subject via related topics such as other side effects of antipsychotic mediation or whether patients had or wanted to have a close personal relationship. These themes are presented in Fig. 1. Some barriers and facilitating factors have bi-directional relationships. For instance, recognition of the importance of a good quality relationship was necessary to raise such “taboo” issues as sexual dysfunction with this patient group, and doing so could potentially enhance the treatment relationship.

## Barriers to recruitment

### Temporal prioritisation

Staff suggested that the decision of whether to talk to their patients about the REMEDY trial was influenced by how long they had had contact with services and how settled their mental state was. Clinicians reported that in managing acutely unwell patients, they prioritised stabilising their mental state over concerns about the side effects of medication. They told us that it was often necessary to leave conversations about side effects of medication until after the patient’s mental health had improved. In navigating the treatment of patients’ who often lacked insight and capacity to make treatment decisions, they found it difficult to discuss sexual dysfunction or the REMEDY trial with their patients.

*“The timing has to be right. You have to look for a way to discuss such a subject with the client or the patient when you have a one to one session and you have to assess or judge the person’s presentation as well.” 1ST025*

*“If someone is psychotic, lacks capacity, can't consent to anything when you're assessing them, these kinds of questions will be put on hold to be explored further when the patient is in a much better mental state and has the capacity to answer them.” 1ST021*

However, once patients were more stable, clinicians suggested that they still had to consider the potential risk of relapse in adjusting the dose or switching medication altogether. They suggested that patients’ mental health would need to be stable over longer periods to be able to tolerate these changes. Some staff reported that patients also preferred to “ride on the side of caution” in sticking with medication that was effective.

“[Patients will say,]*‘No, I don’t want to change my medication, I’m stable on it’ and you can understand that…” 3ST010*

### Contextual demands

Staff often reported that the settings in which they worked, particularly in, but not limited to crisis care, meant that they had limited capacity to engage in the recruitment process or discuss the study with their patients. They reported that it was difficult to keep the trial in mind given that their work often necessitated “spinning multiple plates” and “fighting fires” in terms of managing their patients’ mental states.

*“I don’t know if I’m a bit skewed by being in the Home Treatment Team, seeing people who are acutely psychotic. And almost giving them minimal reason not to take treatment because we might be the ones just starting it or getting them to restart treatment when they’ve had a serious relapse….” 1ST003*

*“…My time tends to be limited with patients because I normally see them at their worst when there is a problem when they're not well so you focus more on the risk assessment.” 1ST019*

High turnover and being consistently understaffed added to the demands of their roles. These service pressures meant that they often did not remember, nor did they have the time to engage in discussion about these side effects or the trial.

“That’s purely maybe because there’s a lack of time, not enough manpower to review side-effects for patients” 1ST029

Some staff suggested that sexual functioning was not a priority for the organisation they worked for or for their service leads. This meant that this trial was not prominent in their minds. Some staff suggested that it would have been helpful to have additional contact, as well as reminders about study and eligibility criteria, from the researchers to facilitate recruitment. However, others felt that they had received adequate support from the team and that the trial had been sufficiently promoted.

*“The researchers have done their best to be present, however ……more presence would have been much more important. So that we have in mind that we need to tackle these questions because as I said not many professionals go in-depth with regard to sexual life.” 1ST030*

*“I think that in the current climate where doctors are very busy it's good that we have reminders that we have people refocusing our attention. But I just wonder about how realistic it will be to expect that people do ask in certain settings when we are just seeing people when they are at their worst.” 1ST019*

### A “taboo” issue

A central theme generated from the staff accounts suggests mutual avoidance of issues around sex in general: staff often don’t ask, and patients can be unwilling to discuss “taboo” subjects. Staff reported that asking about sexual functioning was not part of the clinical culture or routine practice. There was some degree of discomfort, avoidance, or “ignoring” these issues in their consultations with patients. Staff also suggested that these “sensitive”, “personal” and even “taboo” questions could generate feelings of discomfort and embarrassment for patients.

*“I think it’s a two-way thing. The patients tend to be finicky about reporting it because it can be quite personal and we sometimes feel finicky in asking it because it can be quite personal. So both things don’t help. It becomes a hidden thing, that’s something you don’t talk about.” 1ST004*

“….so you see it’s a big taboo for our patients to come us and talk about it. That’s my experience on it.” 1ST017

Staff reported avoiding broaching the subject to prevent embarrassment for both them and their patients. They felt that conversations with patients could entail discussing more sensitive subjects such as masturbation, intercourse, and potentially risky behaviours resulting from sexual disinhibition.

*“…personal embarrassment, or subconsciously or deliberately ignoring* [sexual dysfunction], *I think I’m guilty of [that]”. 3ST007*

Others said that shame associated with mental illness could be further compounded by the shame of sexual dysfunction.

*“This is difficult to say at the moment but based on the people we've had I guess it's probably feeling ashamed to talk about it. Also, the stigma attached with mental illness and also having another problem related with their physical health cascading to sexual dysfunction.” 1ST021*

In particular, staff reported that it could be more difficult to discuss sexual dysfunction between different patient groups and opposite-sex patient-staff treatment relationships. For example, male staff tended to avoid the topic of sex altogether with female patients. Some suggested that it was harder to raise the topic of reduced sexual desire with female patients than it was to discuss erectile dysfunction with younger males.

“It’s something I discuss more with men than women, maybe for women with a male care co-ordinator it’s more difficult to bring up.” 1ST016

Age also seemed to be a consideration for staff, as a few reported that it may not be a prominent concern for older patients, who they suggested, may not be sexually active or were on medication for a prolonged period and therefore accustomed to the sexual side effects.

*“I’ve noticed that a lot of our ladies who are older are very reluctant to talk about it. They don’t want to talk about their sexuality let alone their sexual functioning.” 1ST031*

*“It doesn’t seem to be something that older service users, by that I mean 50 plus, find easy to talk about, maybe that’s because younger people are more sexually active” 1ST016*

Staff frequently raised the issue of the cultural background of their patients. Most staff reported avoiding conversations with patients from more ‘conservative’ backgrounds. Staff suggested that some culture’s view issues of a sexual nature as “taboo”; for example, especially, but not limited, to Asian, Middle Eastern, Muslim, Jewish faiths, and cultures.

“I think if maybe someone who was Muslim, I'd find that difficult because I think a lot of Muslim men, even as a female nurse, I can tell they are holding back from me. They don't seem to see me as equivalent to a male nurse so they are a lot more guarded as a group of people.” 1ST022.

*“It can be considered as a taboo subject for patients especially with different cultural backgrounds. Some patients might not be willing to openly discuss it. Especially if it's a female and you are male staff asking these kinds of questions their husbands or family members who are present might not see it that as acceptable to ask about their sexual life and also if they are experiencing any sexual dysfunction problems.” 1ST021*

### “Reactive” stance

Staff highlighted contrasted side effects like weight gain and extrapyramidal side effects that they could observe or ask the patient about, with sexual dysfunction, where they generally relied on patients self-disclosing.

“We focus on other side effects, and it is easier for us to focus on side effects that we actually see rather than things that we ask.” 1ST019

They suggested that the onus was on patients to be “courage[ous]” and “honest” but acknowledged that it is difficult for patients to be forthcoming. Staff generally adopted a “reactive” stance, rather than taking on a proactive role in exploring these problems themselves.

“….*To me,* [what is of] *importance is what the patient tells me”*. *2ST003*

*“I don’t really feel like it’s appropriate as it’s such a personal and sensitive issue. I’d discuss healthy eating and sleep and topics like that but I wouldn’t just bring up someone’s sexual functioning without them discussing it first.” 1ST016*

Conversely, most staff reported that patients rarely disclosed problems with sexual functioning. Some staff reported that they interpreted this to mean that patients were unaffected by these issues. Others felt that this approach was not ‘best practice’ and stated that they should be proactively asking questions about sexual functioning.

### Difficulty establishing the cause of sexual dysfunction

Staff reported that the process of identifying the various factors that could be causing sexual dysfunction was a complex undertaking. They suggested that they had to test ‘hypotheses’ and rule out other medical or psychological causes, such as age-related decline, depression, diabetes, weight gain, self-image and self-esteem, and other medications.

*“Some sexual issues, it can be related to….*[issues] *of a psychological nature, because the illness might impact their self-confidence…self-esteem or can be of organic cause. So if it’s an organic cause you’re trying to establish the possible factors or co-factors that might be the reason for their sexual dysfunction.” 1ST015*

From their perspective, this could be a difficult process and made assessing eligibility for recruitment to the trial more challenging.

*“It seems for some people that the antipsychotic medications that they are on are clearly linked to their sexual dysfunction. But for others who may have so many side effects including potentially developing diabetes/obesity that may contribute to sexual dysfunction, as well as personal issues ….sometimes it’s difficult to pin down what exactly is causing the dysfunction. Some service users are on antidepressants and antipsychotics so both could be contributing….” 1ST016*

### Caution against unwanted consequences

Staff seemed to have concerns about the unintended consequences of broaching the subject of sexual functioning with patients. A few reported fears about the potential adverse effects that this could have on trust and treatment relationships with patients.

Although staff seemed to avoid the questions with particular patients (e.g. those of the opposite sex, people from minority ethnic communities and people who practised or were believed to practice a religious faith), many across the sample expressed concern about those who were currently experiencing psychotic symptoms and/or paranoid ideation. For example, some staff suggested that patients may misinterpret the motivation behind staff questions and raise complaints if they were asked about sex.

*“*[Patients could become] *agitated, angry, and argumentative and might create unpleasant situations and then you have to avoid it”. 2ST007*

“*Someone said ‘who are you? why do you ask me this? It's none of your business.’”* 1ST023

In addition, staff expressed concerns about broaching the topic with patients who had a history of sexual abuse and those who had been sexually disinhibited. They were concerned that discussion about sex could trigger traumatic memories or increase risk-taking behaviours, putting the patient in potentially harmful situations. Moreover, they feared that some ‘risky’ patients, such as those with deviant sexual fantasies (e.g. paedophilia) or women of childbearing age, who had experience thoughts about harming children, might pose harm to others.

*“We need to be cautious depending on every case….everybody is, as I said, different. We’ve got different patients, and we need to be cautious …on what kind of, part of the life we are going to improve.” 1ST030*

*“When they come on the ward and they are very psychotic, a lot of their time there very paranoid or they’re sexually disinhibited. Which means that if I then start talking about masturbation, I've just encouraged their sexual disinhibition and then they start getting a bit creepy and I have to cut the conversation off.” 1ST022*

Furthermore, other staff reported concerns about the potential impact of conversations about sexual dysfunction on compliance and relapse, particularly those with treatment-resistant conditions. With these patients, achieving stable mental health had often involved a period of trial and error with a range of different drugs. Staff were often reluctant to risk compromising this investment by discussing side effects or changes in medication. Instead, they felt it was better to avoid these questions to maintain fragile treatment relationships and promote compliance with medication.

## Facilitators to discussion of sexual dysfunction

### A significant problem

There appeared to be a consensus among staff that sexual dysfunction was an issue that significantly affects people taking antipsychotic medication, and thus their compliance with it.

*“It's a common significant issue. It's one of the reasons also for non-compliance for stopping medication and relapses. So it is an important issue that we come across.” 1ST020*

*“Maybe it’s one of the main reasons why some patients would want to come off medication….it’s a common complaint that you hear, especially with the younger patients. When they say ‘Oh I don’t want to take this medication, because it’s causing me problems at home.” 1ST023*

However, staff views differed when they were asked how prevalent this problem was among the patients they treated. Many reported that it was a frequent occurrence, but others believed it to be a rare concern. Those who thought it was a common problem, felt that it was often not raised by patients.

“I think it’s very difficult to ascertain, actually. I think it’s a much bigger problem than it appears.” 1ST033

“I think it’s massively under-reported…” 1ST004

“It's difficult to quantify because we probably don't ask as much and patients don't say. So I suspect it's bigger than we think.” 1ST019

### Quality of treatment relationship

It was common for staff to emphasise the importance of building trust and rapport with patients, before they felt they could talk to them about sexual dysfunction. Staff highlighted the intermittent contact they often had with their patients, often during periods of crisis, making it more difficult to build this trust and rapport. Some staff told us that opportunities for developing relationships and having open discussions were greater in inpatient wards and primary care settings.

*“I think it's probably more about having more meaningful time with patients and obviously the more I get to know my patients I'm more likely to ask [about sexual dysfunction]. Because there is also the degree of trust with a doctor and I think sometimes patients may not feel comfortable to talk about this unless they feel they can trust their doctor. “ 1ST019*

*“They will probably raise it with their GP who they trust because some people don't see it as a side effect from the antipsychotics, they see it as a physical problem…. They would prefer to have it from their GP because in their minds, psychiatric services, they are responsible for mental health. My physical health is the responsibility of the GP.” 1ST023*

Some staff reported that once raised, it was possible to have conversations with patients about sexual dysfunction and its potential cause(s). These conversations included a discussion of the risks of changing medication and its possible impact on their current mental state. One staff member suggested that raising the issue with patients could also potentially improve the quality of the treatment relationship.

“If anything it might improve our relationship because then they appreciate that we are acknowledging these problems and trying to help them.” 1ST020

### Strategies for opening dialogue

On those occasions when staff did report having had conversations with patients about sexual functioning, they had often raised the subject indirectly, or by discussing this in the context of other matters.

*“It’s a big issue however it’s difficult to record. So in my career hardly ever do I get patients say*[ing] *they suffer from sexual dysfunction directly. You can get the information indirectly, that it’s making them not have energy, making them unmotivated, making them not have a relationship.” 1ST017*

*“Sometimes I actually just [ask]…’ are you eating well? How’s your sleep been? Do you have a partner? And then whether or not they have a partner you just use that as an opportunity to explore … it’s about exploring those avenues and once they open the topic, they're actually quite happy to talk about it.” 1ST024*

Other staff reported that including such questions as part of a battery of questions during a medication review could be helpful. Many emphasised that developing skills to facilitate these conversations, through formal training as well as provision of appropriate guidance and tools, were necessary to elicit information about these problems.

*“…sometimes it’s difficult to bring it up in general conversation so if you have a form that they can then fill in then you can see whether they’ve ticked it or not and bring it up that way.” 1ST018*

*“ I'm always trying to push people to use objective rating scales so that they are actually asking patients about that in a non-embarrassing way, that they should fill it in.” 1ST026*

## Discussion

We conducted qualitative interviews with 51 front-line staff who were involved in the REMEDY study to examine barriers to recruitment and to try to learn lessons for future research on this topic. The dominant themes in these interviews were that enquires about sexual functioning and sexual side effects of antipsychotic drugs could be embarrassing for patients and staff and were secondary to the more important tasks of maintaining therapeutic relationships and supporting patients to take medication for their mental health. Staff told us that conversations about sexual functioning were particularly difficult with older adults, people of the opposite sex, and people from cultures and religions that they believed were less open to talking about sexuality. In addition, staff told us that they often avoided this topic altogether with people who: had experienced sexual abuse, had paranoid thoughts or had a history of sexual disinhibition.

Many of the staff we interviewed were concerned that talking to patients about sex and sexual functioning could harm their relationship with them. They told us how important it was to develop and maintain a trusting relationship with people with psychosis. They were reluctant to start a conversation with a patient if they felt it could embarrass or offend them. Instead, staff generally waited for patients to bring up the topic themselves. Staff recognised that this was difficult for most patients and often resulted in these problems going unrecognised and untreated.

Staff also told us about factors that made it easier for them to raise the topic of sexual dysfunction and the REMEDY study with their patients. Some were aware that sexual dysfunction is a common side effect of antipsychotic medication and believed it could lead to non-adherence and problems in intimate relationships. These beliefs made it more likely for them to ask patients about sexual dysfunction despite other competing demands on their time. Staff emphasised the importance of having time to develop trust and rapport with patients to make it easier to have conversations about ‘sensitive’ subjects, such as sex. Staff also told us that it was easier to ask people about sexual dysfunction if these questions were preceded by questions about other side effects of medication or whether they had a partner.

Problems in identifying and managing sexual dysfunction have been reported across many medical settings [29–31]. As in the REMEDY study, some of these barriers relate to work pressures and lack of time and other resources. Frontline clinical staff in other healthcare settings also report concerns that patients would not find it acceptable to be asked about sex and sexual functioning [32, 33]. The results of this study suggest that these fears are particularly prominent among staff working with people with psychosis in community settings, where therapeutic relationships can be difficult to maintain and some patients already have poor adherence with medication [34]. While not all people with psychosis want to discuss sex and sexuality with healthcare staff [35], many are happy to discuss these matters [36–38]. Talking to patients about sex also has the potential to improve sexual safety among people with psychosis [39].

Another factor that is widely reported in studies examining barriers to the assessment and management of sexual dysfunction is that staff felt it could open up a discussion about a complicated problem they did not know how best to respond to [29]. In other areas of medicine the ‘PLISSIT’ model has been used to highlight the important role that non-expert clinicians can play in helping people who experience sexual problems [40]. There has relatively little discussion of this approach to people with psychosis and further investigation of this and related models is warranted [41].

Many of those we interviewed told us that enquiring about sexual dysfunction was not part of the clinical culture where they worked. Previous research has also highlighted professional attitudes and organisational culture are barriers to identifying sexual problems among patients [42, 43].

In recent years, progress has been made in changing culture and practice regarding the physical health of people with psychosis. The starting point for this change was recognition of the scale of the problem [44, 45]. This was followed by a series of policy initiatives, financial incentives and public backing of senior leaders and clinicians [46, 47]. Since then the proportion of people with psychosis who receive regular physical health checks and interventions for metabolic side effects of antipsychotic medications has increased [48] and there are early signs that this is beginning to have an impact on people’s health [49, 50].

Numerous reports have highlighted the high prevalence of sexual dysfunction among people with psychosis [1–3]. The findings of this study suggest that front-line staff already recognise that sexual dysfunction is a ‘significant problem’. Many already have and use strategies for ‘opening dialogue’ about sexual dysfunction with their patients, when they feel that they have a good rapport with them. However, the results of this study suggest that changes in organisational culture and clinical leadership at a national and local action will be needed if these ‘hidden’ side effects are to be properly recognised and managed.

The wide range of staff we interviewed from a range of different professional backgrounds, levels of seniority and geographical locations give us confidence that the themes we identified are likely to represent barriers to the assessment and management of sexual dysfunction for people with psychosis more widely. By developing a semi-structured interview in collaboration with people who use and provide services for people with psychosis and sense checking the results with users and providers, we attempted to ensure the credibility of our findings [51]. The main limitation of the study is the absence of qualitative data from patients. We attempted to conduct qualitative interviews with patients who declined to take part in the study, but were unable to collect sufficient material to include in this paper [22]. The study was conducted in four large mental health Trusts in England. We do not know the extent to which the views and experiences of staff working in these organisations compares with staff working with people in other parts of England or in other countries.

The results of our analysis provided some pointers for future research on this topic. Firstly, the impact of adjuvant treatments such as phosphodiesterase inhibitors [52] and aripiprazole [53] may be easier to examine because they do not involve changing the patients existing antipsychotic medication. Secondly, wider use of questionnaires such as the Glasgow Antipsychotic Side-Effect Scale [54, 55], may provide a better way to identify potential participants than relying on front-line staff to raise this matter with potential participants. This would avoid staff having to initiate conversations with patients specifically regarding sexual dysfunction and enable a wider discussion in the context of side effects of antipsychotic drugs. Thirdly, future trials may benefit from trying to recruit patients in primary as well as in secondary care services, where patients are less likely to be in crisis and have great expectation that sexual functioning may be discussed as part of their overall physical health.

## Conclusions

Sexual dysfunction is a distressing and debilitating side effect of antipsychotic medication and can lead to poor adherence and reduced quality of life. A nested qualitative study of staff who participated in a study examining an intervention to manage this problem revealed that many felt uncomfortable discussing sexual dysfunction with their patients and that the management of these side effects was not a priority for the NHS mental health services. In addition, there were concerns that switching antipsychotics could destabilise mental health. Future research is required in this important yet neglected area of medicines management, but it is likely to difficult to complete unless health care organisations and senior clinicians demonstrate greater commitment to improving sexual functioning among people with psychosis.

## Data Availability

Anonymised quantitative data from the REMEDY trial can be obtained from the corresponding author.
